# 3-[2-(3-Phenyl-2-oxo-1,2-di­hydro­quin­oxalin-1-yl)eth­yl]-1,3-oxazolidin-2-one

**DOI:** 10.1107/S1600536813008702

**Published:** 2013-04-05

**Authors:** Ballo Daouda, Mouhamadou Lamine Doumbia, El Mokhtar Essassi, Mohamed Saadi, Lahcen El Ammari

**Affiliations:** a Laboratoire de Chimie Organique Hétérocyclique URAC 21, Pôle de Compétences, Pharmacochimie, Avenue Ibn Battouta, BP 1014, Faculté des Sciences, Université Mohammed V-Agdal, Rabat, Morocco; b Laboratoire de Chimie du Solide Appliquée, Faculté des Sciences, Université Mohammed V-Agdal, Avenue Ibn Battouta, BP 1014, Rabat, Morocco

## Abstract

The di­hydro­quinoxaline ring system of the title mol­ecule, C_19_H_17_N_3_O_3_, is approximately planar [maximum deviation = 0.050 (2) Å], the dihedral angle between the planes through the two fused rings being 4.75 (8)°. The mean plane through the fused-ring system forms a dihedral angle of 30.72 (5)° with the attached phenyl ring. The mol­ecular conformation is enforced by C—H⋯O hydrogen bonds. In the crystal, mol­ecules are linked by weak C—H⋯O hydrogen bonds, forming a three-dimensional network.

## Related literature

For biochemical properties of quinoxaline derivatives, see: Seitz *et al.* (2002 ▶); Monge *et al.* (1993 ▶); Kim *et al.* (2004 ▶); Bailly *et al.* (1999 ▶). For a related structure, see: Caleb *et al.* (2009 ▶).
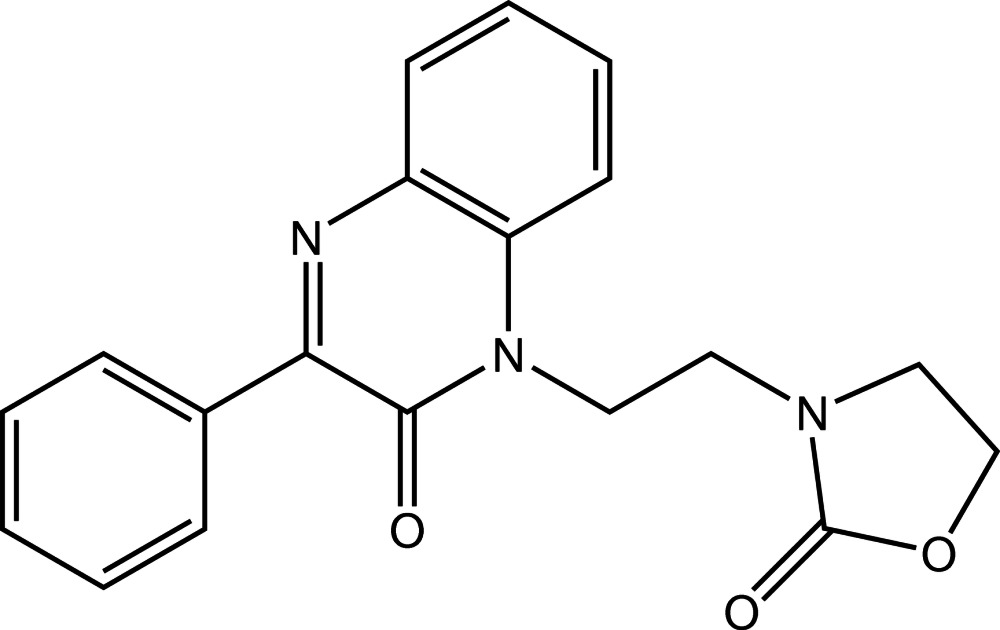


## Experimental

### 

#### Crystal data

C_19_H_17_N_3_O_3_
*M* *_r_* = 335.36
Monoclinic, 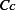

*a* = 9.6314 (5) Å
*b* = 16.6596 (9) Å
*c* = 10.0749 (5) Å
β = 98.097 (3)°
*V* = 1600.46 (14) Å^3^
*Z* = 4
Mo *K*α radiation
μ = 0.10 mm^−1^
*T* = 296 K
0.43 × 0.31 × 0.19 mm

#### Data collection

Bruker X8 APEXII area-detector diffractometer
23600 measured reflections
2249 independent reflections
1897 reflections with *I* > 2σ(*I*)
*R*_int_ = 0.078

#### Refinement

*R*[*F* ^2^ > 2σ(*F* ^2^)] = 0.038
*wR*(*F* ^2^) = 0.097
*S* = 1.03
2249 reflections
226 parameters
2 restraints
H-atom parameters constrained
Δρ_max_ = 0.21 e Å^−3^
Δρ_min_ = −0.23 e Å^−3^

### 

Data collection: *APEX2* (Bruker, 2009 ▶); cell refinement: *SAINT* (Bruker, 2009 ▶); data reduction: *SAINT*; program(s) used to solve structure: *SHELXS97* (Sheldrick, 2008 ▶); program(s) used to refine structure: *SHELXL97* (Sheldrick, 2008 ▶); molecular graphics: *ORTEP-3 for Windows* (Farrugia, 2012 ▶); software used to prepare material for publication: *PLATON* (Spek, 2009 ▶) and *publCIF* (Westrip, 2010 ▶).

## Supplementary Material

Crystal structure: contains datablock(s) I, global. DOI: 10.1107/S1600536813008702/rz5054sup1.cif

Structure factors: contains datablock(s) I. DOI: 10.1107/S1600536813008702/rz5054Isup2.hkl

Supplementary material file. DOI: 10.1107/S1600536813008702/rz5054Isup3.cml

Additional supplementary materials:  crystallographic information; 3D view; checkCIF report

## Figures and Tables

**Table 1:** Hydrogen-bond geometry (Å, °)

*D*—H⋯*A*	*D*—H	H⋯*A*	*D*⋯*A*	*D*—H⋯*A*
C11—H11⋯O1	0.93	2.33	2.860 (3)	116
C17—H17*B*⋯O1	0.97	2.53	3.061 (2)	114
C2—H2⋯O1^i^	0.93	2.42	3.283 (3)	154
C5—H5⋯O3^ii^	0.93	2.50	3.244 (3)	137
C18—H18*B*⋯O1^i^	0.97	2.44	3.367 (3)	159

Symmetry codes: (i) 
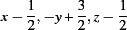
; (ii) 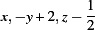.

**Fig. 1.**
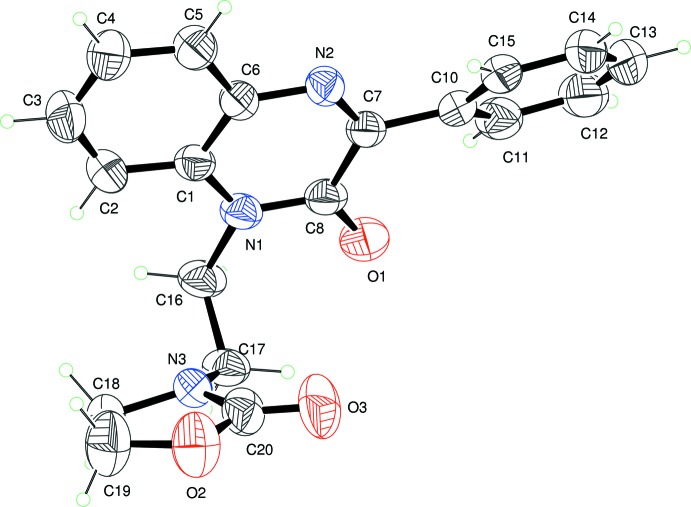
The molecular structure of the title compound with displacement ellipsoids drawn at the 50% probability level. H atoms are represented as small circles or arbitrary radius.

**Fig. 2.**
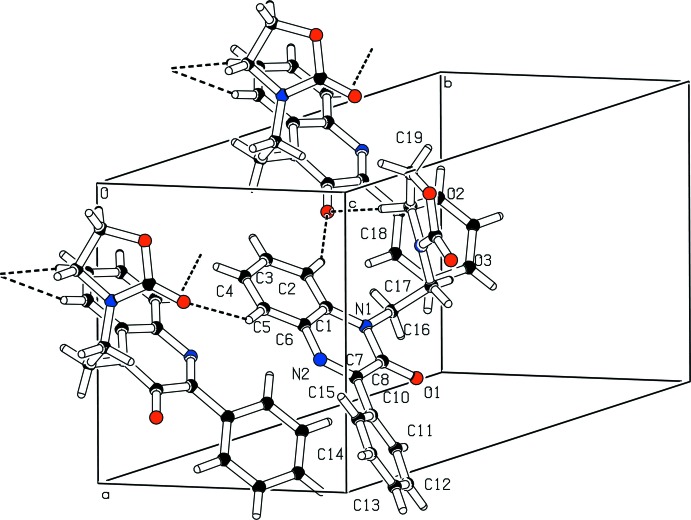
Partial crystal packing of the title compound showing the hydrogen-bonding network (dashed lines).

## supplementary crystallographic information

### Comment

Among the various classes of nitrogen containing heterocyclic compounds, quinoxaline derivatives display a broad spectrum of biological activities (Seitz *et al.*, 2002; Monge *et al.*, 1993; Kim *et al.*, 2004). Quinoxalines play an important role as a basic skeleton for the design of a number of antibiotics such as echinomycin, actinomycin and leromycin. It has been reported that these compounds inhibit the growth of gram-positive bacteria and are also active against various transplantable tumors (Bailly *et al.*, 1999). As a continuation of our research work devoted to the development of substituted dihydroquinoxalin-1-yl derivatives (Caleb *et al.*, 2009) we report in this paper the synthesis and the crystal structure of the title compound.

The two fused six-membered rings (N1/N2/C1–C8) building the molecule of the title compound are approximately planar, the largest deviation from the mean plane being -0.055 (2) Å at C6 (Fig. 1). However, the plane through the two fused rings is slightly folded around the C1–C6 direction as indicated by the dihedral angle between them of 4.75 (8)°. The fused-ring system is linked to the phenyl ring (C10–C15) and to make a dihedral angle of 30.77 (9)°. The oxazolidin cycle (O2/N3/C18/C19/C20) is connected to the fused rings through the C16–C17 chain and build with them a dihedral angle of 68.42 (10)°. The molecular conformation is tabilized by intramolecular C—H···O hydrogen bonds (Table 1).

In the crystal, each molecule is linked to its symmetry equivalent partner by C2–H2···O1, C5–H5···O3 and C18–H18B···O1 non classic hydrogen bonds, forming a three dimensional network as shown in Fig. 2 and Table 2.

### Experimental

In a 100 ml flask 3-phenyl-quinoxalin-2-one (1.25 mmol, 0.28 g) was reacted with dichloroethylamine hydrochloride (2.66 mmol, 0.50 g) in 40 ml of DMF in presence of K_2_CO_3_ (4 mmol, 0.52 g) and tetra-n-butylammonium bromide (0.01 mmol, 0.0032 g). The mixture was brought to reflux in a sand bath with magnetic stirring. The reaction progress was monitored by thin layer chromatography. After evaporation of the solvent under reduced pressure, the residue obtained was chromatographed on silica column (hexane/ethyl acetate 4:6 *v*/*v*). Recrystallization occurred in the same eluent.

### Refinement

H atoms were located in a difference map and treated as riding with C—H = 0.93–0.97 Å, and with *U*_iso_(H) = 1.2 *U*_eq_ (C). In the absence of significant anomalous scattering, the absolute configuration could not be reliably determined and thus 2216 Friedel pairs were merged and any references to the Flack parameter were removed.

### Crystal data

**Table:**

C_19_H_17_N_3_O_3_	*F*(000) = 704
*M**_r_* = 335.36	*D*_x_ = 1.392 Mg m^−^^3^
Monoclinic, *C**c*	Mo *K*α radiation, λ = 0.71073 Å
Hall symbol: C -2yc	Cell parameters from 2249 reflections
*a* = 9.6314 (5) Å	θ = 2.5–29.6°
*b* = 16.6596 (9) Å	µ = 0.10 mm^−^^1^
*c* = 10.0749 (5) Å	*T* = 296 K
β = 98.097 (3)°	Block, colourless
*V* = 1600.46 (14) Å^3^	0.43 × 0.31 × 0.19 mm
*Z* = 4	

### Data collection

**Table:**

Bruker X8 APEXII area-detector diffractometer	1897 reflections with *I* > 2σ(*I*)
Radiation source: fine-focus sealed tube	*R*_int_ = 0.078
Graphite monochromator	θ_max_ = 29.6°, θ_min_ = 2.5°
φ and ω scans	*h* = −13→13
23600 measured reflections	*k* = −23→23
2249 independent reflections	*l* = −13→13

### Refinement

**Table:**

Refinement on *F*^2^	Primary atom site location: structure-invariant direct methods
Least-squares matrix: full	Secondary atom site location: difference Fourier map
*R*[*F*^2^ > 2σ(*F*^2^)] = 0.038	Hydrogen site location: inferred from neighbouring sites
*wR*(*F*^2^) = 0.097	H-atom parameters constrained
*S* = 1.03	*w* = 1/[σ^2^(*F*_o_^2^) + (0.0647*P*)^2^] where *P* = (*F*_o_^2^ + 2*F*_c_^2^)/3
2249 reflections	(Δ/σ)_max_ < 0.001
226 parameters	Δρ_max_ = 0.21 e Å^−^^3^
2 restraints	Δρ_min_ = −0.23 e Å^−^^3^

### Special details

**Table:**

Geometry. All e.s.d.'s (except the e.s.d. in the dihedral angle between two l.s. planes) are estimated using the full covariance matrix. The cell e.s.d.'s are taken into account individually in the estimation of e.s.d.'s in distances, angles and torsion angles; correlations between e.s.d.'s in cell parameters are only used when they are defined by crystal symmetry. An approximate (isotropic) treatment of cell e.s.d.'s is used for estimating e.s.d.'s involving l.s. planes.
Refinement. Refinement of *F*^2^ against all reflections. The weighted *R*-factor *wR* and goodness of fit *S* are based on *F*^2^, conventional *R*-factors *R* are based on *F*, with *F* set to zero for negative *F*^2^. The threshold expression of *F*^2^ > σ(*F*^2^) is used only for calculating *R*-factors(gt) *etc*. and is not relevant to the choice of reflections for refinement. *R*-factors based on *F*^2^ are statistically about twice as large as those based on *F*, and *R*- factors based on all data will be even larger.

### Fractional atomic coordinates and isotropic or equivalent isotropic displacement parameters (Å^2^)

**Table:**

	*x*	*y*	*z*	*U*_iso_*/*U*_eq_	
C19	0.0392 (3)	0.7294 (2)	0.3896 (3)	0.0830 (8)	
H19A	−0.0057	0.7689	0.3270	0.100*	
H19B	−0.0282	0.6876	0.4010	0.100*	
C1	0.43510 (17)	0.88352 (11)	0.25887 (17)	0.0419 (4)	
C2	0.3351 (2)	0.86112 (14)	0.1505 (2)	0.0560 (5)	
H2	0.3144	0.8072	0.1337	0.067*	
C3	0.2676 (2)	0.91989 (16)	0.0689 (2)	0.0646 (6)	
H3	0.2029	0.9048	−0.0043	0.078*	
C4	0.2938 (2)	1.00054 (15)	0.0931 (2)	0.0605 (5)	
H4	0.2473	1.0391	0.0366	0.073*	
C5	0.3888 (2)	1.02337 (13)	0.2009 (2)	0.0509 (4)	
H5	0.4049	1.0776	0.2187	0.061*	
C6	0.46138 (17)	0.96571 (11)	0.28424 (17)	0.0412 (4)	
C7	0.63967 (16)	0.94028 (10)	0.45793 (17)	0.0391 (3)	
C8	0.62007 (19)	0.85246 (11)	0.44060 (18)	0.0431 (4)	
O1	0.69145 (17)	0.80218 (9)	0.50945 (16)	0.0599 (4)	
C10	0.75182 (18)	0.97196 (11)	0.56143 (17)	0.0419 (4)	
C11	0.8763 (2)	0.93092 (13)	0.6025 (2)	0.0548 (5)	
H11	0.8910	0.8805	0.5672	0.066*	
C12	0.9784 (2)	0.96538 (16)	0.6962 (2)	0.0686 (7)	
H12	1.0618	0.9380	0.7232	0.082*	
C13	0.9575 (3)	1.03972 (18)	0.7496 (2)	0.0726 (7)	
H13	1.0261	1.0620	0.8131	0.087*	
C14	0.8350 (3)	1.08113 (15)	0.7089 (2)	0.0611 (5)	
H14	0.8211	1.1315	0.7446	0.073*	
C15	0.73293 (19)	1.04777 (12)	0.61482 (18)	0.0469 (4)	
H15	0.6509	1.0761	0.5869	0.056*	
C16	0.4966 (2)	0.74090 (11)	0.3260 (2)	0.0530 (5)	
H16A	0.5878	0.7160	0.3277	0.064*	
H16B	0.4422	0.7298	0.2394	0.064*	
C17	0.4230 (2)	0.70405 (11)	0.4355 (2)	0.0521 (4)	
H17A	0.4252	0.6461	0.4274	0.063*	
H17B	0.4744	0.7183	0.5221	0.063*	
C18	0.1649 (2)	0.69463 (14)	0.3391 (2)	0.0576 (5)	
H18A	0.1655	0.6365	0.3449	0.069*	
H18B	0.1694	0.7106	0.2472	0.069*	
C20	0.2316 (2)	0.76446 (12)	0.5358 (2)	0.0545 (5)	
N1	0.51490 (16)	0.82872 (9)	0.34210 (15)	0.0436 (3)	
N2	0.56282 (15)	0.99212 (10)	0.38580 (14)	0.0416 (3)	
N3	0.27874 (17)	0.72966 (9)	0.43071 (16)	0.0467 (4)	
O2	0.08945 (18)	0.76632 (12)	0.51602 (18)	0.0767 (5)	
O3	0.2986 (2)	0.79160 (12)	0.63608 (19)	0.0801 (6)	

### Atomic displacement parameters (Å^2^)

**Table:**

	*U*^11^	*U*^22^	*U*^33^	*U*^12^	*U*^13^	*U*^23^
C19	0.0653 (14)	0.110 (2)	0.0686 (17)	0.0044 (14)	−0.0094 (12)	−0.0121 (15)
C1	0.0443 (8)	0.0411 (8)	0.0417 (9)	−0.0065 (7)	0.0114 (7)	0.0006 (7)
C2	0.0574 (11)	0.0558 (12)	0.0537 (11)	−0.0183 (9)	0.0032 (9)	−0.0057 (9)
C3	0.0538 (10)	0.0796 (16)	0.0563 (12)	−0.0125 (11)	−0.0068 (9)	−0.0055 (11)
C4	0.0517 (10)	0.0687 (14)	0.0571 (12)	0.0037 (9)	−0.0063 (8)	0.0067 (11)
C5	0.0475 (9)	0.0475 (10)	0.0559 (11)	0.0025 (8)	0.0012 (8)	0.0029 (9)
C6	0.0410 (7)	0.0402 (9)	0.0424 (9)	−0.0022 (6)	0.0061 (6)	−0.0014 (7)
C7	0.0432 (8)	0.0357 (8)	0.0392 (8)	−0.0023 (7)	0.0090 (6)	0.0009 (7)
C8	0.0517 (9)	0.0353 (8)	0.0435 (9)	−0.0028 (7)	0.0115 (7)	0.0030 (7)
O1	0.0708 (9)	0.0422 (7)	0.0644 (9)	0.0024 (7)	0.0011 (7)	0.0122 (7)
C10	0.0474 (8)	0.0434 (9)	0.0347 (8)	−0.0063 (7)	0.0053 (7)	0.0057 (7)
C11	0.0480 (10)	0.0546 (11)	0.0607 (12)	−0.0009 (8)	0.0040 (8)	0.0120 (9)
C12	0.0506 (11)	0.0777 (17)	0.0732 (16)	−0.0109 (10)	−0.0061 (10)	0.0246 (13)
C13	0.0643 (13)	0.0936 (19)	0.0548 (13)	−0.0329 (13)	−0.0094 (10)	0.0110 (12)
C14	0.0737 (13)	0.0649 (13)	0.0451 (10)	−0.0223 (11)	0.0098 (9)	−0.0058 (10)
C15	0.0530 (9)	0.0487 (10)	0.0398 (9)	−0.0074 (8)	0.0095 (7)	0.0011 (8)
C16	0.0737 (13)	0.0320 (8)	0.0562 (11)	−0.0098 (9)	0.0200 (9)	−0.0034 (8)
C17	0.0621 (11)	0.0377 (9)	0.0573 (11)	−0.0061 (8)	0.0112 (9)	0.0066 (8)
C18	0.0683 (12)	0.0614 (12)	0.0415 (10)	−0.0206 (10)	0.0018 (9)	−0.0036 (9)
C20	0.0671 (12)	0.0394 (9)	0.0548 (12)	0.0007 (8)	0.0007 (9)	−0.0078 (9)
N1	0.0541 (8)	0.0325 (7)	0.0453 (8)	−0.0067 (6)	0.0114 (6)	−0.0006 (6)
N2	0.0444 (7)	0.0377 (7)	0.0420 (7)	−0.0011 (5)	0.0038 (5)	−0.0014 (6)
N3	0.0574 (8)	0.0395 (7)	0.0420 (8)	−0.0105 (6)	0.0025 (6)	−0.0014 (6)
O2	0.0658 (9)	0.0893 (13)	0.0732 (12)	0.0139 (9)	0.0032 (8)	−0.0222 (10)
O3	0.0960 (13)	0.0695 (11)	0.0691 (11)	−0.0031 (9)	−0.0086 (9)	−0.0321 (9)

### Geometric parameters (Å, º)

**Table:**

C19—O2	1.436 (3)	C11—C12	1.388 (3)
C19—C18	1.494 (4)	C11—H11	0.9300
C19—H19A	0.9700	C12—C13	1.376 (4)
C19—H19B	0.9700	C12—H12	0.9300
C1—N1	1.395 (2)	C13—C14	1.378 (4)
C1—C2	1.401 (2)	C13—H13	0.9300
C1—C6	1.409 (2)	C14—C15	1.382 (3)
C2—C3	1.381 (3)	C14—H14	0.9300
C2—H2	0.9300	C15—H15	0.9300
C3—C4	1.383 (4)	C16—N1	1.480 (2)
C3—H3	0.9300	C16—C17	1.521 (3)
C4—C5	1.371 (3)	C16—H16A	0.9700
C4—H4	0.9300	C16—H16B	0.9700
C5—C6	1.397 (3)	C17—N3	1.448 (3)
C5—H5	0.9300	C17—H17A	0.9700
C6—N2	1.383 (2)	C17—H17B	0.9700
C7—N2	1.292 (2)	C18—N3	1.452 (2)
C7—C8	1.482 (2)	C18—H18A	0.9700
C7—C10	1.488 (2)	C18—H18B	0.9700
C8—O1	1.233 (2)	C20—O3	1.207 (3)
C8—N1	1.373 (2)	C20—N3	1.341 (3)
C10—C11	1.391 (3)	C20—O2	1.356 (3)
C10—C15	1.395 (3)		
			
O2—C19—C18	106.27 (19)	C12—C13—C14	120.0 (2)
O2—C19—H19A	110.5	C12—C13—H13	120.0
C18—C19—H19A	110.5	C14—C13—H13	120.0
O2—C19—H19B	110.5	C13—C14—C15	119.9 (2)
C18—C19—H19B	110.5	C13—C14—H14	120.0
H19A—C19—H19B	108.7	C15—C14—H14	120.0
N1—C1—C2	123.65 (16)	C14—C15—C10	120.6 (2)
N1—C1—C6	117.21 (14)	C14—C15—H15	119.7
C2—C1—C6	119.10 (17)	C10—C15—H15	119.7
C3—C2—C1	119.32 (19)	N1—C16—C17	112.34 (17)
C3—C2—H2	120.3	N1—C16—H16A	109.1
C1—C2—H2	120.3	C17—C16—H16A	109.1
C2—C3—C4	121.64 (19)	N1—C16—H16B	109.1
C2—C3—H3	119.2	C17—C16—H16B	109.1
C4—C3—H3	119.2	H16A—C16—H16B	107.9
C5—C4—C3	119.6 (2)	N3—C17—C16	113.57 (17)
C5—C4—H4	120.2	N3—C17—H17A	108.9
C3—C4—H4	120.2	C16—C17—H17A	108.9
C4—C5—C6	120.45 (19)	N3—C17—H17B	108.9
C4—C5—H5	119.8	C16—C17—H17B	108.9
C6—C5—H5	119.8	H17A—C17—H17B	107.7
N2—C6—C5	117.88 (16)	N3—C18—C19	101.73 (18)
N2—C6—C1	122.23 (16)	N3—C18—H18A	111.4
C5—C6—C1	119.82 (16)	C19—C18—H18A	111.4
N2—C7—C8	122.68 (15)	N3—C18—H18B	111.4
N2—C7—C10	117.29 (15)	C19—C18—H18B	111.4
C8—C7—C10	120.03 (15)	H18A—C18—H18B	109.3
O1—C8—N1	120.46 (16)	O3—C20—N3	128.4 (2)
O1—C8—C7	123.54 (17)	O3—C20—O2	121.6 (2)
N1—C8—C7	116.00 (15)	N3—C20—O2	109.92 (17)
C11—C10—C15	118.96 (17)	C8—N1—C1	122.27 (13)
C11—C10—C7	122.94 (17)	C8—N1—C16	115.38 (15)
C15—C10—C7	118.04 (16)	C1—N1—C16	122.29 (15)
C12—C11—C10	119.8 (2)	C7—N2—C6	119.49 (14)
C12—C11—H11	120.1	C20—N3—C17	122.08 (16)
C10—C11—H11	120.1	C20—N3—C18	111.48 (17)
C13—C12—C11	120.6 (2)	C17—N3—C18	122.53 (17)
C13—C12—H12	119.7	C20—O2—C19	109.14 (19)
C11—C12—H12	119.7		

### Hydrogen-bond geometry (Å, º)

**Table:**

*D*—H···*A*	*D*—H	H···*A*	*D*···*A*	*D*—H···*A*
C11—H11···O1	0.93	2.33	2.860 (3)	116
C17—H17*B*···O1	0.97	2.53	3.061 (2)	114
C2—H2···O1^i^	0.93	2.42	3.283 (3)	154
C5—H5···O3^ii^	0.93	2.50	3.244 (3)	137
C18—H18*B*···O1^i^	0.97	2.44	3.367 (3)	159

Symmetry codes: (i) *x*−1/2, −*y*+3/2, *z*−1/2; (ii) *x*, −*y*+2, *z*−1/2.
